# Treatment of Dental Caries When Complicated with an Irritable or Exposed Pulp—Continued

**Published:** 1856-01

**Authors:** James Taylor


### The Dental Register

### OF THE WEST.

VOL. IX. JANUARY, 1856. NO. 2

### Original Essays and Communications.

### TREATMENT OF DENTAL CARIES WHEN COMPLICATED WITH AN LRRITABLE OR EXPOSED PULP—CONTINUED.

BY JAMES TAYLOR.

In our last article several typographical errors occur, one of which completely changes our expression, and which will be found on page 45 and 18th line from the bottom. Instead of "It is not fair to infer," it should read—is it not fair to infer. We might very properly head our present article with this question, how shall we properly diagnose an irritable pulp? We wish for instance to know whether the pulp is in merely an irritable condition or if it is actually exposed. Our first observation is made simply with the eye. The decay appears by its size and the color of the tooth to penetrate near the nerve, and we now interrogate the patient, and we get for answer, the tooth has been *grumbling* for sometime. TIe evidence so far is of a doubtful character. This *grumbling* may be the result of exposure or it may not.

We ask still farther what effect has heat or cold on the tooth? We get for answer either cold or hot fluids give pain, or what is more frequent, cold water gives great pain. Still there is doubt because an irritable pulp having but a thin lamina of inflamed bone as a covering, may give all this pain.

We now take up our instrument and attempt to clear the cavity of some of the extraneous matter which has gotten into it, and the slightest pressure gives pain—the patient exclaims you press on the nerve. Still we may not pronounce the pulp exposed. We may even take a small ball of soft cotton and attempt to dry out the cavity and the pressure of so soft a material gives great pain, and yet the pulp is not exposed. So that the tooth may really have been useless for masticating purposes, for weeks, or even months, it may be highly sensitive, either heat or cold producing pain, and indeed it may have occasionally ached for one to two or three hours at a time, from either pressure of foreign matter in the carious cavity, or from exposure to cold, relief being only obtained by removing the foreign substances from the cavity or by coming to a warm room or getting warm in bed.

All these symptoms we say are not sufficient to diagnose an exposed pulp. They all may, and often do exist, when the pulp is not exposed, but is merely in an irritable condition.

If however the pain becomes continuous and is increased instead of diminished on going to bed, and assumes more of a sharp and then *throbbing* character, we may safely infer that the pulp is either exposed, or has become involved in the general inflammation; and unless this is promply relieved its destruction is inevitable.

We have shown how very deceptive these symptoms may be. For we may often have violent pain as the result of mere sensitive dentine. The vitality of the organ is exalted by the irritation of the disease, and this is particularly the case in the white and brown *variety* of *caries*. We also find the pulp exposed when none of these symptoms have manifested themselves. This occurs often in black caries when it assimulates the brown, or we might perhaps say the brown when it assimulates the black.

It may be asked, is there any positive symptom by which we may determine the condition of the pulp, without removing all the carious matter to see if it is exposed by the disease?

or in other words to see if the dentine has been utterly decomposed to the pulp? We would answer that we have one *unerring* symptom which if present always speaks favorable for the condition of the pulp. We allude to the sensibility of the dentine under the enamel or around the carious opening at a distance from the pulp cavity.

We rely so much on this symptom, seeking here for that vitality which is so essential to the organ, that unless prolonged and severe tooth ache has been present, or the tooth has lost its vital shade of color, we are never content to pass an opinion until we have carefully cut away the caries at some point farthest from the nerve and ascertained the condition of the dentine. We prefer an exalted sensibility to an obtunded one; the latter we regard as the exhausted vital energy so much dreaded in typhoid fever; the former as perhaps denoting an excessive nervous irritability, yet giving assurance of a vital power and energy on which it is far more safe to rely. We have the means at hand to subdue the one, but where shall we find the agent to restore the other?

We are prepared now to consider the causes producing an irritable condition of the pulp. The first and most direct cause is caries. This is a diseased condition of the dental organs resulting in decomposition of the tooth substance, but it may be asked does this always induce irritability of the pulp? We answer, not—but it depends very much on the agent inducing the disease, and the general condition of the secretions of the mouth. In some cases the cause of caries appears to completely obtund the sensibility of the dentine as rapidly as decomposition takes place, while in others we have increased sensibility, and this increased sensibility may be induced by an acid condition of the secretions of the mouth, and yet the direct cause of decay be the same as when the sensibility is obtunded.

We regard it important to properly determine if the secretions are of such a character as to induce irritation. If such be the fact, due attention should be given to them with proper neutralizing remedies.

Inflammation of the pulp of one tooth may and does without doubt, produce irritation of the nerves of all those teeth on the same side of the mouth, and sometimes of all the teeth. This irritation will be particularly felt in the teeth when caries approximates the pulp. This is manifest from the fact that an aching tooth often involves other teeth much decayed, and so much is this the case, that patients often lose sight of the one that is really producing the difficulty.

Every practitioner is familiar with such cases—often being required to extract teeth from the wrong jaw. Inflammation of the gums will also induce irritation of the pulp when near exposed. We have really a two-fold influence operating to induce irritation: first, the inflammation of the gums; and second, the acid condition thus induced in the secretions.

Imperfect dental operations, and general constitutional irritability, will with many other causes excite irritation in the pulp but thinly covered by dentine; extremes of heat or cold is a very common cause.

With this view of the case before us, we are prepared to treat it understandingly, regarding inflammation of the gums and tooth ache, as direct sources of irritation; the first thing to be done in the treatment of irritable pulps, preparatory to filling the teeth, is the removal of all aching teeth, which upon examination we find should be extracted to put the mouth in a healthy condition, all roots of teeth which are sources of irritation—the salivary calculus which keeps the gums inflamed. This as far as possible may be done at the first sitting, and then such detergent and astringent washes as may be desirable to allay irritation and restore health. If constitutional disturbance is manifest some general remedies should be prescribed to meet the case, generally a mild cathartic will be sufficient, or if much nervous excitement is present, an anodyne may be administered.

The most prominent difficulties having been thus overcome, our attention should next be directed to the organ or organs we may wish to preserve.

This preparatory treatment generally modifies very much the acid condition of the secretions of the mouth, and we are now prepared to pay attention to the direct effect of the cari ous matter which covers the pulp itself, and which we must regard also as an irritant.

We are satisfied that the treatment thus far directed, will often completely change the condition of the organs, we may wish to preserve. This will be noted by the less amount of pain, and the ease with which cold and hot fluids may be brought in contact with the teeth. Yet although much has been accomplished in the preparation for filling such teeth, there is often still much to be done to prepare difficult cases of this kind for filling.

Our attention is then next directed to the carious portion of the tooth to be removed. This should be carefully removed excavating first that which lays farthest from the nerve. In doing this if pain is induced, we prefer only removing a part at one sitting, and then introduce a small pledget of cotton moistened with creosote dipped in tannin, covering the whole, or sealing up the tooth with wax or gum mastic. This operation we repeat every day or two until the entire decayed portion is removed and the cavity will bear the introduction and compacting of cotton or some such substance without any special pain.

We have frequently resorted to the use of collodion, but this generally induces more pain when first applied and we have not found it so reliable as the creosote and tannin, which by the caustic or antiseptic property of the one and the astringent of the other appears to exactly meet the exigency of such cases.

It would be well to bear in mind that all arsenical preparations are here inadmissible. The close proximity of the pulp prevents the use of those remedies which might be available in the treatment of mere irritable dentine. Chloride of zinc which at times is a very efficient remedy for the treatment of inflamed dentine, we also discard in cases of irritable pulp and agree with Prof. G. Watt, who in speaking of the use of this article as an escharotic for inflamed dentine, remarks, "Of course it should never be used when it is practicable to restore the inflamed part to its normal condition." And he further remarks, that "the subjacent tissues are left in a healthier condition after its use than after nitrate of silver and some other caustics, but the loss of substance is greater."

It is this loss of substance and the danger of inducing inflammation of the pulp which forbids the use of any caustic preparation, having an affinity to the lime of the tooth.

We regard the creosote as not of the same character as the caustic potassa, nitrate of silver, chloride of zinc, etc. The direct action of the agent (creosote) on inflamed dentine is rather difficult satisfactorily to explain. It is regarded by Webster, Ure and other authors as an antiseptic and powerful stimulant. Its direct action on the mucous tissue appears to be caustic—its direct effect on inflamed dentine is to relieve pain, and not to diminish the sensibility by any caustic property. That its antiseptic property may be useful in preventing decomposition of that lamina of bone which is inflamed, and which is the only covering to the pulp, we think is highly probable. We see no reason why antiseptic remedies should not be resorted to in such cases for such an object, as well as to prevent disorganization of other tissues of the economy when laboring under disease.

Considering the true pathology of the disease under consideration, and the known antiseptic property of this remedy, we then prescribe it for a two-fold effect; first, its penetrating soothing influence, and second, for its antiseptic action on that portion of dentine, which we wish to preserve from disorganization.

Pathologically there are several questions in relation to the condition of this lamina of bone which covers the pulp, which are important.

Regarding caries as strictly of chemical origin, can we, by the removal of that part of the tooth already decomposed, be certain that we have removed all the agent inducing disease. Does this decomposed structure hold in solution the chemical agent for the further destruction of the tooth? has not the chemical change which has taken place in these constituents made of it a neutral agent? Such appears to be the law of chemical action. If so the lamina of bone beneath this decomposed structure has received already a charge of decomposing fluid, or it is passed from the secretions of the mouth through this carious matter to this part.

Any given portion of an acid cannot take up more than its quantum of lime; when this is done all action ceases. In the brown variety of caries we find that generally the lime is completely removed. The carious matter is at least of a cartilaginous structure and the lime is being removed as fast as the chemical agent can effect it. This view of the subject enables us to have some idea of the kind of an acid inducing the disease. It should also point out a suitable neutralizing agent. If for instance, by proper tests we detect traces of acetic acid in the saliva, wash out the carious cavity with the solution of some substance with which this acid will unite more readily than lime. If the muriatic or nitric acids, use also an agent with which these will unite more readily than with the base of the teeth.

In the treatment then of irritable pulps we regard the washing of the cavity out, from time to time, as we remove the caries as of some consequence, thereby neutralizing the agent causing the tooth's decomposition. Tepid water used freely is a powerful neutralizer, and when this is used no deleterious compound is left in the cavity. We have often used a solution of the sub-carbonate of soda or carbonate of magnesia.

If this lamina of bone which covers the pulp has already received a charge of decomposing fluids their first action appears to be to induce inflammation of the dentine, and then a disintegration of its parts, neutralizing agents must arrest this action or the increased vitality induced, arouse nature to resist and restore, or the amount of acid received will spend its force and not cease to act until it has become neutralized. Chemically considered, the latter would appear to be the effect, and yet will not a living tissue resist this action when a dead one could not? Can the calcareous elements be removed, say chemically from the tubuli of which the dentine are composed and those tubes themselves be not destroyed? or can a deposition of lime take place repairing the loss? Nature may be insufficient for this task. Yet she goes to work to protect herself and not only deposits new bone for a covering but condenses and hardens that which is not destroyed.

It should be borne in mind, that in the treatment of the pulps of teeth thus far considered, we have not an utter exposure, and also that we have to treat a lamina of inflamed dentine. The treatment is to preserve the vitality of the tooth, and this the only covering to the pulp.

We regard this covering, although diseased, as a far better covering than any other which can be substituted in its place.

The question might very properly be asked, should this covering be retained even if its vitality cannot be preserved? We answer, by all means; unless its constituents are disintegrated or inflammation supervenes in the pulp requiring its destruction. If the latter takes place, it will be denoted by increased pain, soreness of the tooth when struck upon, &c.

The treatment proposed will generally diminish the sensibility in from four to ten days, and when such is the case and the carious portion can be removed, the tooth is ready for filling.

We have still, however, one resource left us after all; these remedies have been used, and the tooth is still too tender for a solid and permanent filling. It is this: Remove all the softened caries, preparing all the walls of the cavity for a plug, but leaving over the nerve all of the partially decomposed dentine which cannot be removed without much pain; wash out the cavity well with warm water, then take of Hill's soft filling material and cut a cap of the same to lay over the pulp cavity; fit this carefully over the same when in its hard state; then with a sufficient quantity of the same, properly warmed, fill up the entire cavity.

This excludes all foreign matter, and being a non-conductor, prevents the extremes of heat or cold from affecting injuriously the tooth. This should not, however, be done until the general irritability has passed off. This we regard as a temporary operation, giving time for the pulp organ to throw out a deposition of bony matter sufficient for a proper covering. We leave this in from one to six months, and within the last week took out one which had been in for two years. In this latter case the tooth was too tender for masticating purposes for over six months. On its removal we found the pulp alive, and but little decomposed dentine in the bottom of the cavity. When this was removed, which was dry and powdery, the dentine was hard and glass-like. In this case, as in most of such cases, the inflamed dentine was well preserved, and hence the agent inducing decomposition had all been removed or well neutralized.

For these temporary fillings we prefer this material and this course to any of the gum preparations, which would have to be renewed frequently.

For sometime a few years back we frequently used the Collodion, applying it with enough cotton to fill the cavity, also relying on it more or less to subdue the irritability of the dentine and pulp; but the pain on its first introduction appeared to render it less reliable than the Cresote and Tannin.

We come now to speak of that condition of the pulp called exposed, or when the caries has reached this organ, the dentine having been utterly destroyed or decomposed. We shall first speak of the treatment we adopt for its preservation, and then when this is impracticable, the means left us still for preserving the tooth by its destruction.

We have not thought it necessary to give any special directions, as it regards the filling of the teeth after the preparatory treatment which we have given. We would, however, simply remark that the method of filling is just the same as in those cases where this treatment has not been necessary, except that when the teeth are still sensible to the extremes of heat or cold. we apply over the nerve a non-conducting material. This is generally made of Hill's soft filling, sometimes oil silk, asbestos, &c., &c.; but of late we have discarded almost entirely all these materials but the former, because we have found it more easily adapted to the bottom of the cavity and equally as reliable.

